# Impact of middle leaders’ competencies on teachers’ technology acceptance in Malaysian TS25 schools

**DOI:** 10.1371/journal.pone.0332614

**Published:** 2025-09-19

**Authors:** Jeunhan Yip, Kenny S. L. Cheah, Wen Fen Beh

**Affiliations:** 1 Faculty of Education, University of Malaya, Kuala Lumpur, Malaysia; 2 Faculty of Education, University of Malaya, Kuala Lumpur, Malaysia; 3 Faculty of Creative Arts, University of Malaya, Kuala Lumpur, Malaysia; RMIT University, VIET NAM

## Abstract

This study examines how middle leaders’ competencies influence teachers’ technology acceptance in Malaysian TS25 primary schools. The research combines the Middle Leaders’ Competency Model for Education 4.0 (MLCMEdu4.0) and the Teachers’ Technology Acceptance Model (TTAM). Using a cross-sectional survey, data were gathered from 302 teachers in TS25 schools in Kuala Lumpur and analyzed using Structural Equation Modeling (SEM). Results show a significant positive relationship (β = 0.599, p < .001), with middle leaders’ competency accounting for 36% of the variance (R² = .36) in teachers’ technology acceptance. Critical thinking was identified as the most vital middle leadership competency in Education 4.0, followed by entrepreneurial skills and collaboration. Among factors influencing teachers’ technology acceptance, perceived ease of use, perceived usefulness, and facilitation conditions were most impactful. This study highlights the critical role of middle leaders in supporting technology acceptance, especially in Malaysia’s educational reform context. It highlights the need for targeted professional development programs and clear role definitions for middle leaders to enhance their influence on technology acceptance. Future studies should explore these dynamics across varied educational contexts and adopt longitudinal designs to assess the long-term effects of middle leadership on technology acceptance.

## 1. Introduction

The landscape of education is rapidly evolving in response to technological advancements and the needs of the Fourth Industrial Era. In Malaysia, this transformation is guided by the Malaysia Education Blueprint (2013–2025) and initiatives like the *Transformasi Sekolah 2025* (TS25) program, which aim to modernize the education system and equip students to tackle the challenges of the 21st century [1–3]. The TS25 program is a school-improvement initiative providing targeted support through structured modules on topics ranging from leadership empowerment to digital tools. A core feature of this initiative is the direct capacity-building provided to school leadership teams by a network of Master Trainers (MTs) and an Educational Leadership and Instructional Team (ELIT) [2]. These expert coaches facilitate workshops and deliver on-site coaching to strengthen instructional leadership and foster professional learning communities among both senior and middle leadership teams. While this formal, top-down support structure is designed to guide school transformation, the practical implementation of reforms like technology integration still relies heavily on the influence and competency of school-level leaders. Central to this transformation is the integration of technology in education, aligning with the principles of Education 4.0, which highlights personalized learning, digital competency, and the development of both technical and soft skills [4,5].

Middle leaders in education organization hold a unique position between senior management and teachers [6–8]. Their potential to influence teaching practices and drive educational change is increasingly recognized [9], however, their effect on teachers’ acceptance of technology remains unclear, particularly within Malaysian primary schools. This gap is particularly notable given the emphasis on distributed leadership in recent educational reforms, which aims to empower leaders at all levels to drive school improvement and innovation. Furthermore, although considerable research has been carried out on principals’ technology leadership, there is a scarcity of research examining how middle leaders, through distributed leadership practices, can facilitate and enhance teachers’ technology acceptance. This oversight is significant, as middle leaders typically engage in more direct and frequent interactions with teachers [7,10,11], which enables them to exert a more immediate and profound impact on daily classroom practices and the implementation of technology.

The development of Education 4.0 and the rapid pace of technological change have also created new challenges for middle leaders [11–13]. They are expected to not only understand and use new technologies themselves but also to guide and support teachers in incorporating these technologies into their teaching methods. However, the competencies required for middle leaders to effectively fulfill this role in the context of Education 4.0 are not well defined or understood. Additionally, the implementation of the TS25 program as part of Wave 3 of the Malaysia Education Blueprint presents a unique context for studying middle leadership and technology acceptance. While this program emphasizes technology transformation in schools, the specific mechanisms through which middle leaders influence teachers’ technology adoption and use within this framework remain unclear.

This study aims to address these research gaps by examining the impact of middle leaders’ competencies on teachers’ technology acceptance in TS25 primary schools in Malaysia. By integrating the Middle Leaders’ Competency Model (MCMEdu4.0) with the Teachers’ Technology Acceptance (TTA), this research provides an approach for analyzing the dynamics of educational leadership and technology integration in the context of Malaysia’s educational reforms.

As Malaysian schools strive to address the demands of Education 4.0 and implement the TS25 program, understanding the role of middle leaders in facilitating technology acceptance becomes crucial for successful educational reform. This study’s findings have direct implications for policy-making, professional development programs, and school improvement strategies in Malaysia and other countries facing similar educational transformations.

## 2. Literature review

### 2.1 Middle leadership in educational management

Middle leadership has emerged as a critical component in educational management, facilitating the connection between senior leadership and classroom teachers. In the Malaysian context, middle leaders, often referred to as head of department, subject heads, *“Ketua Panitia”* play a pivotal role in school operations and improvement initiatives [14]. Middle leaders typically hold dual roles as both teachers and administrators, placing them in a unique position to influence both instructional practices and organizational processes. Their responsibilities often include curriculum implementation, teacher supervision, and departmental management [6–8].

In the setting of TS25 schools, middle leaders have been given increased responsibilities. They are expected to implement professional support for improving teacher quality in curriculum, co-curriculum, and student affairs [2,15]. This expanded role underscores the importance of middle leaders in driving school transformation and implementing national education policies at the ground level.

However, middle leaders in school face several challenges in fulfilling their roles effectively:

i. Time constraints: Balancing teaching duties with leadership responsibilities often leads to work overload [16,17]
ii. Lack of senior leadership support: Some middle leaders report insufficient backing from senior management, hindering their effectiveness [18,19]
iii. Inadequate preparation and training: Many middle leaders feel underprepared for their leadership roles, indicating a need for more comprehensive professional development [20,21]
iv. Role conflict and ambiguity: The dual nature of their position can lead to unclear expectations and conflicting demands [18,22]

Despite these challenges, middle leadership practices have shown significant potential in enhancing school effectiveness. Research suggests that effective middle leaders can positively influence teacher professional development, curriculum implementation, and overall school improvement [9,10,23]. In the rapidly evolving landscape of Education 4.0, middle leaders are increasingly expected to lead technological integration and pedagogical innovation [24]. However, their capacity to influence teachers’ technology acceptance and use remains an area requiring further investigation, particularly in the Malaysian context. This overview of middle leadership highlights its importance in educational management while also pointing to the need for further research on how middle leaders’ competencies impact various aspects of school improvement.

### 2.2 Middle leaders’ competency model in education 4.0

The rapid changes in education, especially within Education 4.0, have prompted a reevaluation of the competencies essential for effective educational leadership. While there are established models for principals and senior leaders, there is increasing recognition of the need for competency frameworks specifically tailored to middle leaders, given the challenges of technological advancement and educational transformation. Current research on leadership competencies for Education 4.0 remains limited, with a primary focus on general leadership skills rather than the specialized competencies required for middle leaders to address the demands of the fourth industrial revolution [15,25].

This study seeks to address the limitation by applying the School Leadership Competency Model for Education 4.0 (SLCMEduc4.0) developed by [13]. While initially designed for general school leaders, the model offers a comprehensive framework suitable for middle leaders as well. The SLCMEduc4.0 outlines nine key competencies essential for effective leadership in the context of Education 4.0.

i. ***Digital Dexterity***: This competency involves the ability to adapt quickly to new digital technologies and leverage them effectively in educational settings [26]. For middle leaders, this might involve guiding teachers in the integration of digital tools in their teaching practices [11,27].
ii. ***Leading for Learning***: This competency focuses on the leader’s ability to create and sustain a practice of ongoing learning and development. Middle leaders are pivotal in cultivating learning communities and advancing teacher development. [28,29].
iii. ***Collaboration***: In the context of middle leadership, this competency involves facilitating cooperation among teachers, departments, and external stakeholders to enhance educational outcomes [10,24].
iv. ***Emotional Intelligence***: This competency is crucial for middle leaders in managing relationships, navigating conflicts, and providing support to both teachers and students. [30,31]
v. ***Critical Thinking***: Middle leaders need to utilize critical thinking skills in problem-solving, decision-making, and in evaluating the effectiveness of educational programs and practices [3,32].
vi. ***Entrepreneurial***: This involves the ability to innovate, take calculated risks, and identify opportunities for improvement within the school system [21,27].
vii. ***Decision-making and Problem-solving***: Middle leaders often face complex situations that require effective decision-making skills, balancing various stakeholder interests [12,33].
viii. ***Communication and Ethics***: Effective communication and adherence to ethical standards are crucial for middle leaders in their interactions with teachers, students, parents, and senior management [15,34].
ix. ***Management and Administration***: This competency involves the ability to efficiently manage resources, implement policies, and oversee departmental operations [6,8].

While the SLCMEduc4.0 provides a comprehensive framework, its application to middle leaders in the Malaysian context remains an area for further exploration. how these competencies manifest in middle leadership roles and their impact requires further investigation. Understanding how these competencies can be developed and applied in the context of TS25 schools could provide valuable insights for enhancing middle leadership effectiveness and, consequently, improving technology integration in Malaysian education.

### 2.3 Teachers’ technology acceptance in educational settings

The integration of technology in education has become vital, especially with Education 4.0 and the acceleration of digital transformation due to events like the COVID-19 pandemic [35]. Understanding the factors that impact teachers’ technology acceptance is crucial for the effectiveness of educational technology efforts. The Technology Acceptance Model (TAM) [36], has been frequently applied to explain and predict technology adoption, including in education. Over time, TAM has been adapted to better reflect the complexities of technology acceptance in educational contexts [37,38]. This study considers several key factors influencing teachers’ technology acceptance.

i. ***Perceived Usefulness***: Refers to how much teachers believe that incorporating technology will enhance their work quality, such as enhancing teaching effectiveness, student engagement, or administrative tasks. PUseful is a strong predictor of technology adoption in educational settings [38].
ii. ***Perceived Ease of Use***: Refers to teachers’ belief that using technology will be effortless. User-friendly interfaces and technical support improve PEaseU, which influences technology acceptance directly and indirectly [36,39]
iii. ***Facilitation Conditions***: Describes teachers’ perception of having sufficient organizational and technical support, such as hardware, software, and internet access, to use technology effectively [40,41]
iv. ***Subjective Norm***: Involves the social pressure teachers feel to use or not use technology, influenced by leadership, peers, or the school community, which varies based on school culture [39,42].
v. ***Technology Self-Efficacy***: Refers to teachers’ confidence in their ability to use technology effectively, with higher self-efficacy leading to greater adoption of technology in education [43,44].
vi. ***Technological Pedagogical Content Knowledge***: A framework that highlights the integration of technology, pedagogy, and content knowledge, linked to effectiveness of technology use in teaching [45,46].

## 3. Conceptual framework of middle leaders’ competency model in education 4.0 (MLCMEdu4.0) impacts on teachers’ technology acceptance (TTA)

**Fig 1:**
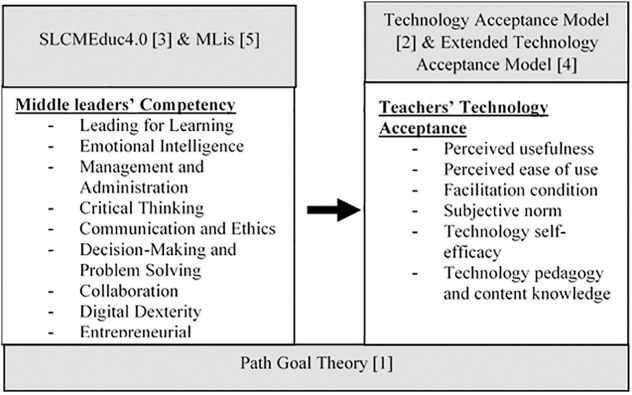
Conceptual Framework of MLCMEdu4.0 and TTA.

This study integrates the Middle Leaders’ Competency Model (MLCMEdu4.0), based on SLCMEduc4.0 [13] and MLis [6], with the Technology Acceptance Model [36] and its extended version [47]. This framework (as shown in Fig 1) examines the relationship between middle leaders’ competencies and teachers’ technology acceptance within the context of Education 4.0. Grounded in Path Goal Theory, this study examines how middle leaders facilitate technology adoption among teachers, conceptualizing mechanisms that potentially enhance technological integration in educational settings.

## 4. Methodology

### 4.1 Research design

This study adopted a quantitative research design using Structural Equation Modelling (SEM) to examine the impact of Middle Leaders’ Competency in Education 4.0 (MLCMEdu4.0) on Teachers’ Technology Acceptance (TTA). The SEM framework incorporated both measurement and structural models, allowing simultaneous assessment of construct validity and the hypothesized relationships between variables.

The final model comprised 11 latent variables and 16 observed variables. First-order factors of MLCMEdu4.0 were treated as latent variables when modelled in relation to MLCMEdu4.0, but as observed composites in relation to their indicators. Similarly, TTA components were treated as observed variables. Parceling was employed as a modelling strategy to create more parsimonious models, improve indicator reliability, and reduce correlated measurement errors [48]. Given that both instruments were based on established theoretical dimensions, parceling provided a stable and theoretically coherent basis for testing the model.

### 4.2 Sample size justification

Using the [49] formula, a target sample size of 302 teachers was determined from the population of 1,464.

Although SEM-specific recommendations, such as [50] calculator, suggest a sample size of 195 for detecting a medium effect size and a minimum sample size of 552 for model structure, researchers selected a sample of 302 from a total population of 1,464. This decision was influenced by practical constraints, including time and logistics, which limited the size of our sample despite the acknowledged need for a larger one due to the model’s complexity. Sample of 302 still meets several critical guidelines:

- It exceeds the critical sample size of 200 for stable parameter estimates [51,52].
- It adheres to [53] ‘rule of 10’ (160 required).
- It surpasses [54] recommendation of 15 cases per indicator (240 required).
- It satisfies [55] guideline of 5 cases per parameter (220 required for our 44 parameters).

Researcher acknowledge the limitations and potential biases of sample size. While a larger sample might have enhanced statistical power and lowered the likelihood of Type II errors, time and logistical constraints limited our ability to fully represent the total population of 1,464 teachers. These constraints may affect the generalizability of our findings. Nevertheless, the sample of 302 achieves an appropriate balance between statistical rigor and the practical constraints of educational research, facilitating a robust SEM analysis

### 4.3 Population and sampling

This study employed a proportional stratified random sampling technique to ensure representation across the population of 1,464 teachers from 34 Cohort-5 TS25 schools in the Federal Territory of Kuala Lumpur. These schools were distributed across three *Pejabat Pendidikan Wilayah* (PPW): *Bangsar Pudu* (17 schools), *Keramat* (9 schools), and *Sentul* (8 schools). A proportional stratified random sampling technique was employed, with each school serving as a stratum. The number of teachers selected from each school was proportional to its total teaching staff. Within each school, participants were selected using simple random sampling.

To ensure the target sample size for each school was met, a replacement protocol was used. In instances where a selected teacher declined to participate or was unavailable, a new teacher was randomly selected from the remaining pool of eligible teachers within the same school. This process was repeated until the pre-determined proportional sample for each school was achieved, resulting in a final, fully constituted sample of 302 participants.

The final allocation was as follows: *Bangsar Pudu* – 142 teachers (17 schools), *Keramat* – 86 teachers (9 schools), and *Sentul* – 74 teachers (8 schools), reflecting each school’s proportional staff size. All 302 invited teachers agreed to participate, resulting in a 100% cooperation rate across districts (*Bangsar Pudu* 142/142; *Keramat* 86/86; *Sentul* 74/74). Since all invitees participated, the replacement protocol (whereby non-respondents would be replaced by randomly selected alternates from the same school) was not required.

### 4.4 Demographic profile

The demographic characteristics of the sample are presented in Table 1.

**Table 1:** Demographic Characteristics of the Respondents.

Category		Frequency	Per cent (%)
**Gender**	Male	56	18.5
	Female	246	81.5
**Age**	24-30	37	12.3
	31-35	54	17.9
	36-40	46	15.2
	41-45	94	31.1
	46-50	33	10.9
	51-55	25	8.3
	56-60	13	4.3
**Highest Education Level**	Diploma	9	3.0
	Bachelor’s	261	86.4
	Master’s	32	10.6
**Years of Teaching Experience**	1-5	36	11.9
	6-10	59	19.5
	11-15	77	25.5
	16-20	70	23.2
	21-25	25	8.3
	26-30	23	7.6
	More than 30	12	4.0

To contextualize the sample, its demographic profile was compared with national teacher population statistics reported by the Ministry of Education Malaysia (2025). Two observations follow. First, the proportion of female teachers in the sample (81.5%) modestly exceeds the national average (≈72%). Second, the distribution of educational qualifications is closely aligned: 97% of respondents hold at least a bachelor’s degree, comparable to the national figure of 94% university graduates. Overall, although female teachers are slightly overrepresented, the sample is broadly representative of the Malaysian teaching workforce in terms of qualifications, supporting the generalizability of the study’s findings.

### 4.5 Ethical considerations and data collection

Ethical clearance was obtained from the University Malaya Research Ethics Committee (UMREC ref UM.TNC2/UMREC_2290, 31 May 2023), along with permission from the relevant District Education Offices and the principals of all 34 schools. The researcher visited each school to meet with the principal, confirm arrangements, and personally brief the selected teachers. These sessions explained the purpose of the study, the voluntary nature of participation, participants’ rights, and the consent form, as well as the instructions for completing the questionnaire.

The data collection process adhered to ethical guidelines, obtaining necessary permissions from ERAS and UMREC. Questionnaires were distributed to the selected sample, prioritizing participant anonymity, informed consent, and data confidentiality.

Questionnaires and consent forms were completed voluntarily within an agreed time frame, typically three days and sealed in individual envelopes to maintain confidentiality. The researcher returned to each school to collect the completed materials directly from the principal or a designated staff member. No personal identifiers were recorded, and all procedures adhered to the ethical principles outlined in the 2013 revision of the Declaration of Helsinki.

### 4.6 Research instruments

Two main instruments were utilized: Middle Leaders’ Competency Model questionnaire, adapted from the SLCMEduc4.0 [13], and a Teachers’ Technology Acceptance questionnaire, derived from various Technology Acceptance Model (TAM) studies [36,47]. Both instruments employed a 7-point Likert scale and were structured to measure multiple constructs related to middle leaders’ competency and teachers’ technology acceptance respectively.

Instrument items were reviewed by a panel of four experts in educational management, planning, and policy from the University of Malaya. The experts assessed each item for clarity, construct relevance, and contextual appropriateness for Malaysian TS25 primary schools. Based on their evaluations, the instrument achieved an excellent Scale-Level Content Validity Index (S-CVI/Ave) of 0.963, surpassing the recommended benchmark of 0.90 [56]. Minor wording revisions were made in response to their feedback to improve clarity and contextual fit.

An initial pilot test with school teachers (from a different sample) confirmed high internal consistency across all scales (Cronbach’s α > 0.83) but also revealed some redundancy within several subscales. To improve efficiency, conceptually overlapping items were merged, resulting in a more concise instrument. A second pilot test verified that the revised version maintained excellent reliability, giving confidence in the suitability of the questionnaire for the main study.

### 4.7 Data analysis procedure

Data analysis was conducted using AMOS for Confirmatory Factor Analysis (CFA) and SEM. The process involved the following steps:

i. A normality test was conducted to verify multivariate normality using two methods:
  - Checking that item skewness falls within +/-2, with a critical region not exceeding 8.0
  - Ensuring kurtosis is within +/-10, with a critical region also not exceeding 8.0.
ii. The first-order measurement model fit was assessed to evaluate how well observed variables (items) aligned with hypothesized first-order factors (latent variables). The following Table 2 shows the fit indices used and their thresholds:
iii. Construct reliability was measured to ensure the items consistently measured the underlying constructs. This was done using Cronbach’s Alpha and Composite Reliability (CR), with reliability scores above 0.70 generally considered acceptable.
iv. Construct validity was examined through two aspects:
  - Convergent validity was checked to ensure items measuring the same construct were highly correlated, using Average Variance Extracted (AVE) with a threshold of > 0.50.
  - Discriminant validity was tested to ensure different constructs were distinct, using the Fornell-Larcker criterion and Heterotrait-Monotrait (HTMT) ratio.
v. A second-order measurement model fit was tested to determine if first-order factors loaded onto a higher-order factor. Similar fit indices to those used in the first-order model were employed to assess the fit of the second-order structure.

**Table 2:** Model Fit Index Threshold.

Fit Indices	Recommended Value	Source (s)
P	Insignificant	[57]
Model Chi-Square Test (CMIN/df)	3-5	Less than 2 [58] to 5 [59]
Comparative Fit Index (CFI)	>.95	[60]
Tucker Lewis Index (TLI)	>.95	[60]
Standardized Root Mean Square Residual (SRMR)	<.08	[60]
Root Mean Square Error of Approximation (RMSEA)	<.06	[60]

Lastly, the researcher examined the relationship between MLCMEdu4.0 and TTA through structural model testing, employing the parceling approach. This method involved combining individual indicators into composite scores to simplify the model while still capturing the key relationships between the constructs.

## 5. Quantitative findings: confirmatory factor analysis and structural model assessment

To enhance the clarity of findings, researchers present the results in three main sections: (5.1) the measurement model of MLCMEdu4.0, (5.2) the measurement model of TTA, and (5.3) the structural model showing the relationship between MLCMEdu4.0 and TTA. Each section includes detailed explanations of factor loadings, model fit indices, and their interpretations.

### 5.1 MLCMEdu4.0 measurement model

#### 5.1.1 Normality assessment of MLCMEdu4.0.

In this study, normality assessment of the MLCMEdu4.0 data revealed that all values fell within acceptable ranges, confirming normal distribution, as shown in Table 3.

**Table 3:** Normal Distribution of MLCMEdu4.0.

Aspect	Observed Range	CR Range	Acceptable Range	Status
Skewness	−1.023 to −0.066	−7.285 to −0.467	+/-2 (CR < 8)	Normal
Kurtosis	−0.786 to 1.547	−2.625 to 5.505	+/-10 (CR < 8)	Normal

**The First-order Measurement Model of MLCMEdu4.0.** The measurement model demonstrates robust factor loadings across all constructs, indicating high validity of the items used to operationalize each concept. The results are as follows:

i. Digital Dexterity (DD) displays factor loadings between 0.85 and 0.89, indicating a strong correlation between the assessed behaviors and digital competency, reinforcing the effectiveness of the measurement.
ii. Leading for Learning (LoL) presents loadings between 0.82 and 0.86, demonstrating that leadership behaviors related to teacher development and effective resource utilization are well-represented within this construct.
iii. Collaboration (Collab) shows factor loadings in the range of 0.84 to 0.90, confirming that activities indicative of fostering teamwork is effectively captured by the measurement items.
iv. Emotional Intelligence (EmoInt) displays loadings from 0.87 to 0.90, indicating a strong correlation between the measured variables, including empathy and conflict resolution skills, and the latent construct.
v. Critical Thinking (CritT) exhibits loadings ranging from 0.84 to 0.91, suggesting that the items effectively measure cognitive processes such as information evaluation and pattern recognition.
vi. Entrepreneurial (Entre) construct demonstrates loadings between 0.82 and 0.87, indicating a robust relationship between the measured variables, including adaptability to change, and the underlying concept.
vii. Decision-making and Problem-solving (DMPS) presents loadings from 0.86 to 0.89, suggesting that the items effectively capture the ability to make decisions under pressure and solve complex problems.
viii. Communication and Ethics (CET) shows high factor loadings ranging from 0.87 to 0.91, indicating strong measurement validity for both communication skills and ethical behavior.
ix. Management and Administration (MA) exhibits loadings between 0.81 and 0.87, demonstrating good representation of skills related to resource management and policy implementation.

**Fig 2:**
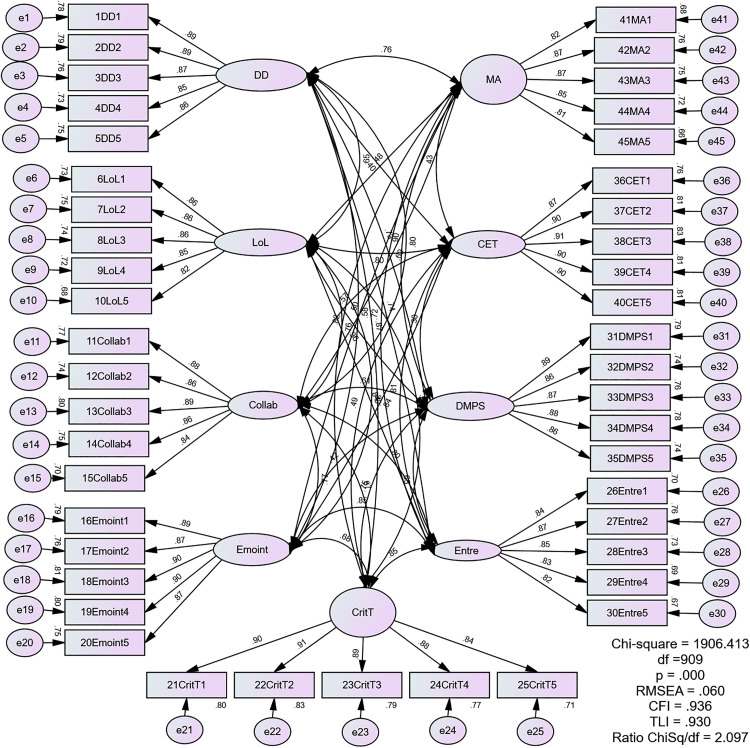
The First-order Measurement Model of MLCMEdu4.0.

To assess the model’s overall goodness of fit, researchers employed multiple fit measures including CMIN/df, CFI, TLI, SRMR, and RMSEA. Our findings demonstrated that all indices (CMIN/df = 2.097, CFI = 0.936, TLI = 0.930, SRMR = 0.0405, RMSEA = 0.060) were within their respective common acceptance levels, as shown in Fig 2. These results collectively suggest that MLCMEdu4.0 fits the data well.

#### 5.1.3 Construct reliability.

The reliability analysis of the study’s constructs yielded consistently strong results as shown in Table 4. All constructs demonstrated high reliability, with both Cronbach’s alpha and Composite Reliability values exceeding the critical threshold of 0.70. This indicates that the items within each construct are internally consistent and effectively measure their intended concepts. The high Composite Reliability values further confirm that each construct is reliably measured as a whole. These strong reliability scores give researchers confidence in the measurements’ accuracy and consistency. This provides a solid basis for further analysis.

**Table 4:** Construct Reliability of MLCMEdu4.0.

Constructs	No. of Items	Cronbach Alpha (α)	Composite Reliability (C.R.)
DD	5	0.941	0.941
LoL	5	0.929	0.930
Collab	5	0.937	0.938
CritT	5	0.947	0.946
Emoint	5	0.946	0.948
MA	5	0.924	0.925
DMPS	5	0.940	0.941
CET	5	0.953	0.954
Entre	5	0.925	0.926

#### 5.1.4 Construct validity.

This study employed a comprehensive approach to establish construct validity, examining both convergent and discriminant validity. A crucial consideration in this analysis is the study’s theoretical framework, which posits the 9 middle leader competencies as first-order factors of a single higher-order construct. Consequently, while these factors must be statistically distinct (discriminant validity), non-trivial correlations between them are theoretically expected. Our goal was to balance the need for statistical clarity with the theoretical coherence of the higher-order model.

Convergent validity, the degree to which indicators of the same construct share variance, was confirmed using the Average Variance Extracted (AVE). As shown in Table 5, all constructs exceeded the recommended 0.50 threshold, indicating that the measurement items were highly correlated with their intended latent factors.

**Table 5:** Convergent Validity of MLCMEdu4.0.

Construct	Average Variance Extracted (AVE)
DD	0.762
LoL	0.725
Collab	0.751
CritT	0.779
Emoint	0.784
MA	0.712
DMPS	0.761
CET	0.805
Entre	0.714

The researchers attempted to assess discriminant validity using a method that compares each construct’s Average Variance Extracted (AVE) to its relationships with other constructs. This approach, known as the Fornell-Larcker criterion, suggests that for valid discrimination, a construct’s AVE should exceed the squared correlation it has with any other construct, or alternatively, the square root of a construct’s AVE should be greater than its correlations with other constructs. However, when applied to their data, this method failed to fully establish discriminant validity. Several constructs, including Digital Dexterity (DD), Leading for Learning (LoL), Collaboration (Collab), Critical Thinking (CritT), Decision-making and Problem-solving (DMPS), and Entrepreneurial (Entre), did not meet the required criteria as shown in the Table 6 through bolded values.

**Table 6:** MLCMEdu4.0 Fornell & Lacker Criterion for Discriminant Validity.

	DD	LoL	Collab	CritT	Emoint	MA	DMPS	CET	Entre
**DD**	**0.873**								
**LoL**	0.655	**0.852**							
**Collab**	0.799	0.760	**0.867**						
**CritT**	0.875	0.652	0.872	**0.883**					
**Emoint**	0.581	0.877	0.765	0.679	**0.886**				
**MA**	0.757	0.481	0.695	0.817	0.505	**0.845**			
**DMPS**	0.733	0.558	0.609	0.748	0.417	0.900	**0.872**		
**CET**	0.598	0.803	0.573	0.492	0.841	0.430	0.301	**0.897**	
**Entre**	0.738	0.840	0.797	0.849	0.853	0.719	0.651	0.807	**0.844**

Therefore, the more rigorous Heterotrait-Monotrait (HTMT) ratio was applied. This analysis revealed a high correlation between the Decision-making and Problem-solving (DMPS) and Management and Administration (MA) constructs, with an HTMT ratio of 0.903, which is at the limit of acceptable thresholds. (See Table 7).

**Table 7:** MLCMEdu4.0 Heterotrait-Monotrait (HTMT) ratio for Discriminant Validity.

	DD	LoL	Collab	CritT	Emoint	MA	DMPS	CET	Entre
**DD**									
**LoL**	0.654								
**Collab**	0.805	0.762							
**CritT**	0.877	0.650	0.874						
**Emoint**	0.587	0.880	0.767	0.683					
**MA**	0.758	0.477	0.700	0.818	0.506				
**DMPS**	0.735	0.360	0.615	0.758	0.421	0.903			
**CET**	0.398	0.806	0.575	0.487	0.842	0.421	0.297		
**Entre**	0.739	0.845	0.807	0.850	0.861	0.717	0.654	0.810	

Upon further inspection, the DMPS1 item was identified as the primary source of the high correlation, suggesting potential conceptual redundancy or item-level misfit. To improve the integrity of the measurement model, a decision was made to remove this item. This decision represents a deliberate trade-off: by removing the item, we slightly narrow the content coverage of the DMPS construct, but in exchange, we achieve a cleaner model with unambiguous discriminant validity, which is critical for testing the structural model. We prioritized measurement integrity on the basis that the remaining items adequately preserved the theoretical core of the construct. After this single adjustment, all HTMT ratios fell below the 0.90 threshold, successfully establishing discriminant validity for all constructs (Table 8). This final adjusted model, having demonstrated both convergent and discriminant validity while balancing statistical rigor with theoretical coherence, was used for further analysis.

**Table 8:** MLCMEdu4.0 Heterotrait-Monotrait (HTMT) ratio for Discriminant Validity after Revision.

	DD	LoL	Collab	CritT	Emoint	MA	DMPS	CET	Entre
**DD**									
**LoL**	0.654								
**Collab**	0.805	0.762							
**CritT**	0.877	0.650	0.874						
**Emoint**	0.587	0.880	0.767	0.683					
**MA**	0.758	0.477	0.700	0.818	0.506				
**DMPS**	0.727	0.355	0.605	0.756	0.414	0.896			
**CET**	0.398	0.806	0.575	0.487	0.842	0.421	0.297		
**Entre**	0.739	0.845	0.807	0.850	0.861	0.717	0.654	0.810	

#### 5.1.5 The second-order measurement model of MLCMEdu4.0.

**Fig 3:**
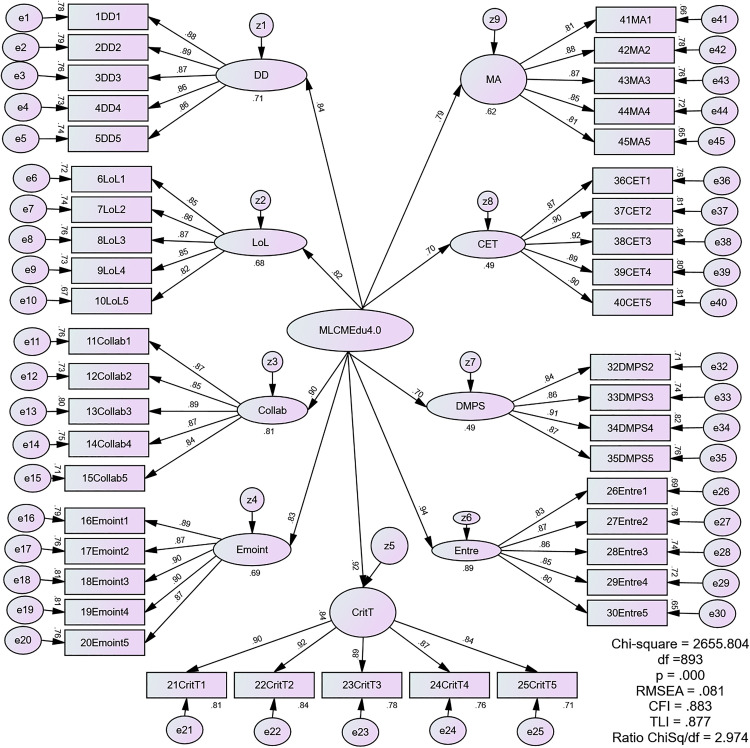
The Second-order Measurement Model of MLCMEdu4.0.

An initial higher-level examination of MLCMEdu4.0, as shown in Fig 3, revealed inadequate model fit. The fit indices were unsatisfactory: CMIN/df = 2.974, CFI = 0.883, TLI = 0.877, SRMR = 0.1025, and RMSEA = 0.081. To improve the model, a re-estimation was performed based on modification indices. This process involved relaxing the initial model’s assumption of forced orthogonality, where all measurement error is presumed to be random and uncorrelated. To improve the model’s fit, with a particular focus on enhancing TLI, CFI, and Standardized RMR, a re-estimation process was carried out. This process involved correlating error terms based on the highest modification index (MI) for each iteration, acknowledging that any single change could affect the entire model. The process unfolded in several steps: first, z9 and z7 were correlated (MI = 141.152), followed by z4 and z8 (MI = 90.308), then z8 and z6 (MI = 60.349), z2 and z5 (MI = 50.341), and finally z2 and z8 (MI = 23.573).

**Fig 4:**
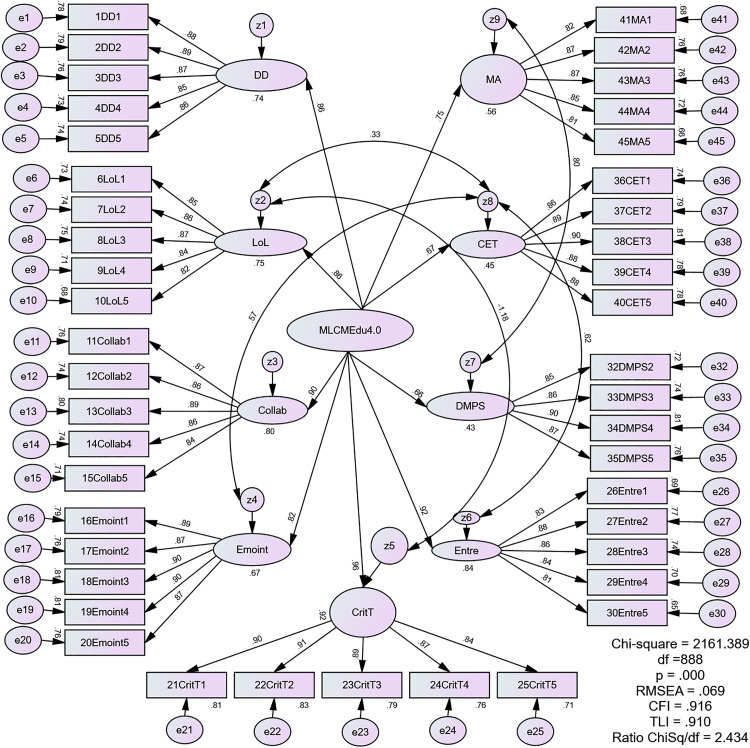
The Re-estimated Second-order Measurement Model of MLCMEdu4.0.

By systematically correlating the error terms of several first-order factors (specifically z9 with z7, z4 with z8, z8 with z6, z2 with z5, and z2 with z8), the revised model reinstates systematic associations that were previously suppressed. In essence, the superior fit of the final model is achieved because it now accounts for nuanced relationships and shared variance between specific leadership competencies that are not fully explained by the overarching MLCMEdu4.0 construct. While this data-driven approach provides a better representation of the sample data, we acknowledge it increases model complexity and must be interpreted with caution. Fig 4 presents the final re-estimated model which exhibited a good fit with the following improved indices: CMIN/df = 2.434, CFI = 0.910, TLI = 0.916, SRMR = 0.0794, and RMSEA = 0.069. Complementing this visual representation, Table 9 presents a summary of the CFA results for the higher-order construct of the MLCMEdu4.0 measurement model.

**Table 9:** Summary of CFA Results for Higher Order Construct of Measurement Model MLCMEdu4.0.

	Standardized Estimates	t-values
**Relationship to Higher Order Construct of Middle Leaders’ Competency**		
Digital Dexterity → MLCMEdu4.0	0.858	**
Leading for Learning → MLCMEdu4.0	0.864	13.439
Collaboration → MLCMEdu4.0	0.897	14.41
Emotional Intelligence → MLCMEdu4.0	0.818	13.666
Critical Thinking → MLCMEdu4.0	0.961	15.257
Entrepreneurial → MLCMEdu4.0	0.918	14.572
Decision-making and Problem-solving → MLCMEdu4.0	0.655	10.728
Communication and Ethics → MLCMEdu4.0	0.668	11.012
Management and Administration → MLCMEdu4.0	0.746	11.911

Model Fit Statistics (x^2^ = 2161.389, df = 888, Ratio x^2^/df = 2.434; CFI = 0.916, TLI = 0.910, SRMR = 0.0794, RMSEA = 0.069).

** = Items constrained for identification purposes.

### 5.2 Teachers’ technology acceptance measurement model

#### 5.2.1 Normality assessment of TTA.

For the normality test on the TTA data, researchers confirmed that all values fell within acceptable parameters, verifying a normal distribution pattern, as shown in Table 10.

**Table 10:** Normal Distribution of TTA.

Measure	Observed Range	CR Range	Acceptable Range	Status
Skewness	−1.004 to −0.174	−7.146 to −1.239	±2 (CR < 8)	Normal
Kurtosis	−0.458 to 1.771	−2.027 to 6.303	±10 (CR < 8)	Normal

#### 5.2.1 The first-order measurement model of TTA.

The measurement model demonstrates robust factor loadings across all constructs, indicating strong construct validity and reliable operationalization of the concepts under study.

- Perceived Usefulness (PUseful): Factor loadings range from 0.82 to 0.93, highlighting a strong connection between the measured items and the perceived benefits of technology.
- Perceived Ease of Use (PEaseU): Loadings fall within the range of 0.87 to 0.90, demonstrating strong convergent validity for items assessing the perceived user-friendliness of technology.
- Subjective Norm (SN): Exhibits high loadings of 0.89 and 0.91, indicating robust measurement of social influence factors in technology acceptance.
- Facilitation Condition (FC): Displays loadings from 0.85 to 0.89, signifying strong representation of organizational and technical infrastructure support for technology use.
- Technology Self-Efficacy (TSE): Presents strong loadings of 0.86 and 0.88, indicate a solid measurement of individuals’ confidence in using technology.
- Technological Pedagogical Content Knowledge (TPACK): Demonstrates a range of loadings from 0.79 to 0.92, indicating good construct representation of the complex interplay between technology, pedagogy, and content knowledge.

The consistently high loadings between 0.75 and 0.93 demonstrate that the items reliably capture the intended constructs, making them well-suited for assessing various aspects of teachers’ technology acceptance.

**Fig 5:**
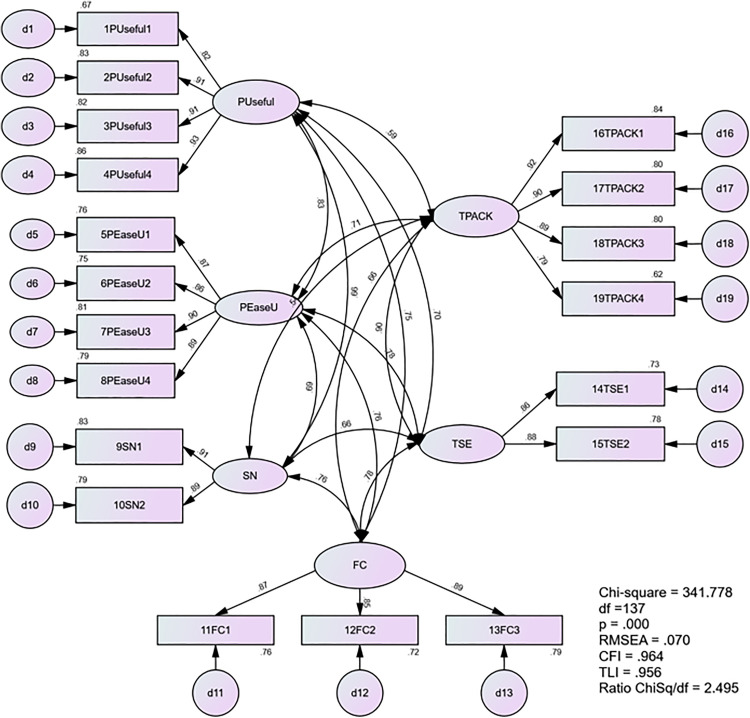
The First-order Measurement Model of TTA.

In this study, the analysis demonstrated that all model fit indices (CMIN/df, GFI, CFI, TLI, SRMR, and RMSEA) met their respective commonly accepted thresholds as shown in Fig 5. The specific values obtained were: CMIN/df = 2.495, CFI = 0.964, TLI = 0.956, SRMR = 0.0311, and RMSEA = 0.070, indicating a strong fit between the proposed model and the observed data.

#### 5.2.3 Construct reliability.

The TTA demonstrates strong reliability across all constructs as shown in Table 11. Cronbach’s Alpha and Composite Reliability scores exceed the 0.70 benchmark for each construct, indicating high internal consistency and measurement accuracy.

**Table 11:** Construct Reliability of TTA.

Constructs	No. of Items	Cronbach Alpha (α)	Composite Reliability (C.R.)
PUseful	4	0.937	0.940
PEaseU	4	0.932	0.933
SN	2	0.892	0.892
FC	3	0.903	0.902
TSE	2	0.861	0.861
TPACK	4	0.925	0.929

#### 5.2.4 Construct validity.

For TTA, researchers assessed convergent validity using Average Variance Extracted (AVE) for each construct. Convergent validity was confirmed using Average Variance Extracted (AVE): Table 12 shows that all constructs exceeded the 0.50 threshold.

**Table 12:** Convergent Validity of TTA.

Constructs	Average Variance Extracted (AVE)
PUseful	0.796
PEaseU	0.776
SN	0.806
FC	0.754
TSE	0.757
TPACK	0.765

For discriminant validity, initially using the Fornell-Larcker criterion. However, this approach didn’t fully establish discriminant validity, as two constructs didn’t meet the required criteria as shown in Table 13: Technology Self-efficacy (TSE) and Technology Pedagogy and Content Knowledge (TPACK). For these constructs, the square root of their AVE was less than their correlation with each other.

**Table 13:** MLCMEdu4.0 Fornell & Lacker Criterion for Discriminant Validity.

	PUseful	PEaseU	SN	FC	TSE	TPACK
PUseful	**0.892**					
PEaseU	0.832	**0.881**				
SN	0.659	0.693	**0.898**			
FC	0.750	0.764	0.755	**0.868**		
TSE	0.696	0.782	0.657	0.783	**0.870**	
TPACK	0.590	0.709	0.545	0.664	0.904	**0.875**

As a result, the researchers adopted an alternative method, the Heterotrait-Monotrait (HTMT) ratio. When assessed using the HTMT ratio, all ratios were less than the limit of 0.90. This result successfully established discriminant validity for all constructs in the Teachers’ Technology Acceptance (TTA) as shown in Table 14.

**Table 14:** MLCMEdu4.0 Heterotrait-Monotrait (HTMT) ratio for Discriminant Validity.

	PUseful	PEaseU	SN	FC	TSE	TPACK
PUseful						
PEaseU	0.837					
SN	0.666	0.692				
FC	0.745	0.759	0.744			
TSE	0.693	0.786	0.658	0.782		
TPACK	0.592	0.714	0.547	0.658	0.896	

This final model, having demonstrated both convergent and discriminant validity through the AVE and HTMT analyses respectively, was then used for further analysis in the study.

#### 5.2.5 The second-order measurement model of TTA.

**Fig 6:**
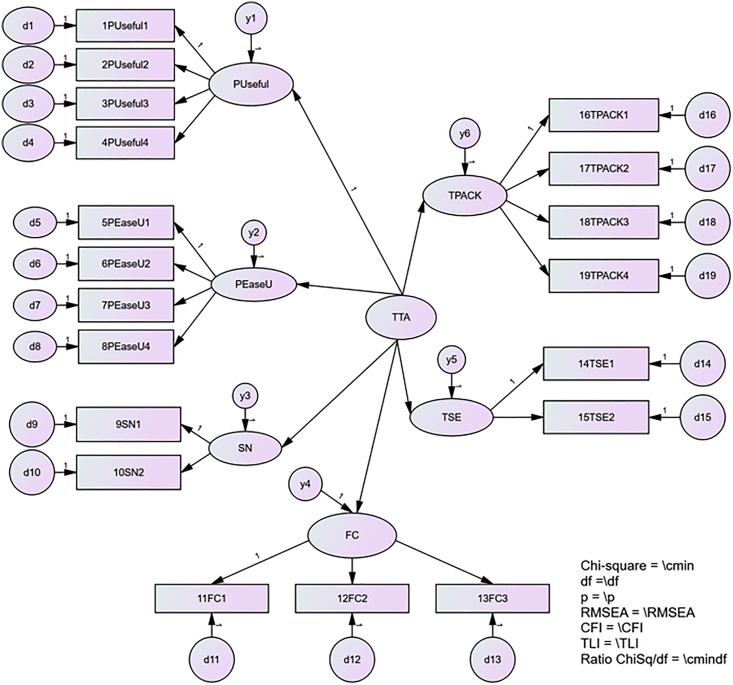
The Second-order Measurement Model of TTA.

An examination of the second-order TTA model, as illustrated in Fig 6, initially revealed an inadequate fit, with an RMSEA value exceeding the desired threshold: CMIN/df = 3.273, CFI = 0.942, TLI = 0.933, SRMR = 0.0538, and RMSEA = 0.087. To address this, the model was re-specified based on the highest modification index (MI = 83.890), which suggested a significant correlation between the error terms of Technology Self-Efficacy (TSE) and TPACK. This adjustment relaxes the model’s initial assumption of forced orthogonality and reinstates a theoretically meaningful association that was previously suppressed. Substantively, this suggests that a teacher’s confidence in using technology (TSE) and their pedagogical knowledge of how to apply it (TPACK) share a unique relationship not fully accounted for by the general ‘Technology Acceptance’ factor.

**Fig 7:**
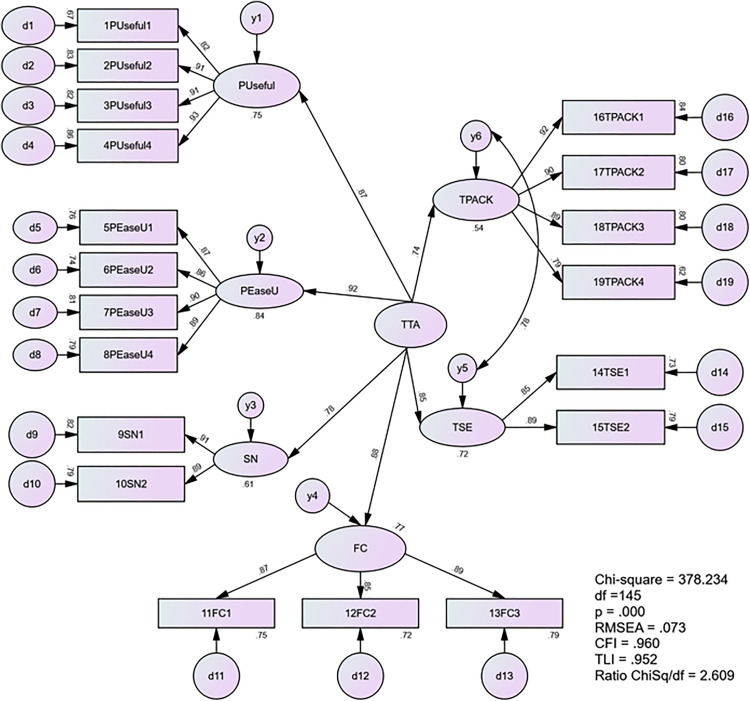
The Re-estimated Second-order Measurement Model of TTA.

Following this adjustment, Fig 7 presents the final re-estimated model which demonstrated a significantly improved fit. The new indicators were as follows: CMIN/df = 2.609, CFI = 0.960, TLI = 0.952, SRMR = 0.0392, and RMSEA = 0.073. These improved metrics indicating a good fit between the model and the data. Table 15 provides a comprehensive overview of the CFA outcomes for the higher-order construct within the TTA measurement model.

**Table 15:** Summary of CFA Results for Higher Order Construct of Measurement Model TTA.

	Standardized Estimates	t-values
**Relationship to Higher Order Construct of** **Teachers’ Technology Acceptance**		
Perceived Usefulness → Teachers’ Technology Acceptance	0.868	**
Perceived Ease of Use → Teachers’ Technology Acceptance	0.915	13.976
Subject Norm → Teachers’ Technology Acceptance	0.782	12.213
Facilitation Condition → Teachers’ Technology Acceptance	0.876	13.314
Technology Self-efficacy → Teachers’ Technology Acceptance	0.847	12.668
Technology Pedagogy and Content Knowledge → Teachers’ Technology Acceptance	0.736	11.838

Model Fit Statistics (x^2^ = 378.234, df = 145, Ratio x^2^/df = 2.609; CFI = 0.960, TLI = 0.952, SRMR = 0.0392, RMSEA = 0.073).

** = Items constrained for identification purposes.

### 5.3 Structural model of MLCMEdu4.0 and TTA

In this study, researchers employed structural equation modeling (SEM) using AMOS to investigate the relationship between middle leaders’ competency and teachers’ technology acceptance.

**Fig 8:**
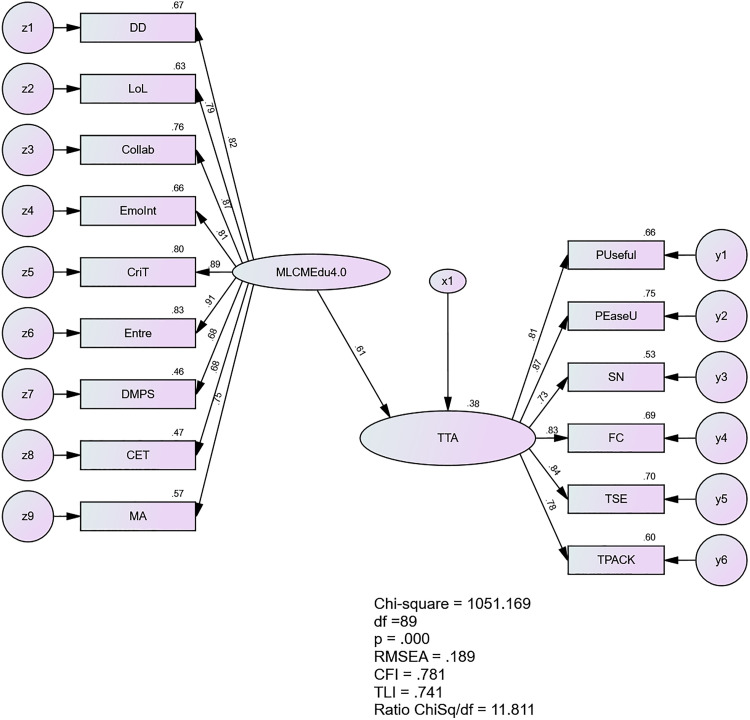
The structural model of MLCMEdu4.0 and TTA.

Initially, the structural model doesn’t yield a good fit for the data as shown in Fig 8: CMIN/df = 11.811, CFI = 0.781, TLI = 0.741, SRMR = 0.0777, and RMSEA = 0.189. To improve the model’s fit, the researchers systematically correlated variables, starting with those having the highest modification indices (MI). The first error terms correlated was z9 and z7 (MI = 144.408), then followed by z8 and z4 (MI = 92.661), y5 and y6 (MI = 73.607), z8 and z6 (MI = 63.413), z5 and z2 (MI = 50.835), z8 and z2 (MI = 24.060), z8 and z2 (MI = 24.060), z4 and z2 (MI = 27.567), z6 and z4 (MI = 19.636), z6 and z2 (MI = 24.061), z9 and z8 (MI = 13.951), z7 and z2 (14.902), z4 and z3 (MI = 15.213), y1 and y2 (MI = 13.108).

**Fig 9:**
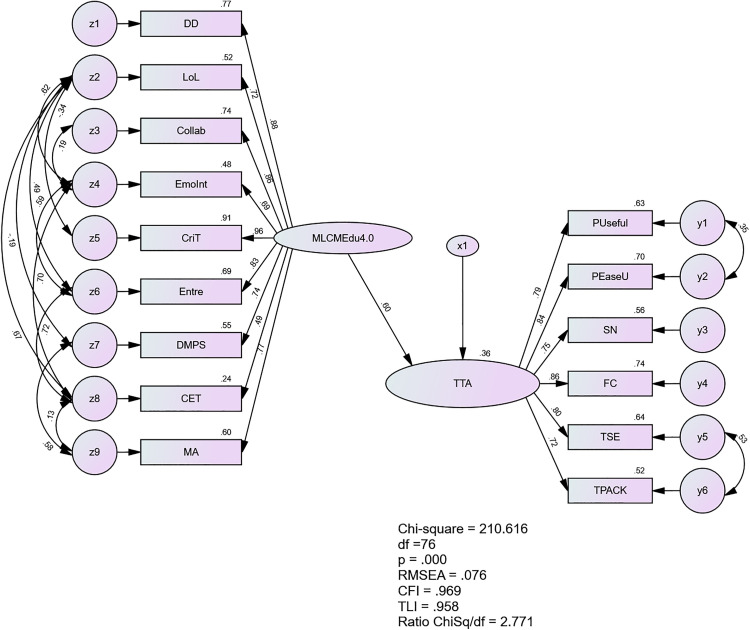
The Re-estimated Structural Model of MLCMEdu4.0 and TTA.

After re-estimation according to the modification indexes, model fit was able to achieve, with CMIN/df = 2.771, 1CFI = .969, TLI = .958, SRMR = .0508 and RMSEA = .076. The model after re-estimation is show in Fig 9.

The results as shown in Table 16 revealed a significant positive relationship between middle leaders’ competency and teachers’ technology acceptance (β = .599, p < .001). This moderately strong relationship accounted for 36% of the variance in teachers’ technology acceptance. These findings highlight the crucial role of middle leaders’ competency in influencing teachers’ adoption of technology in educational settings. The results suggest that enhancing middle leaders’ competencies could be an effective strategy for improving technology acceptance among teachers.

**Table 16:** Model Fit Indices and Hypothesis Results of Structural Equation Model for MLCMEdu4.0 and TTA.

Hypothesized Relationship	Standardized Estimates	t-value	p-value	Decision
Middle Leaders’ Competency → Teachers’ Technology Acceptance	0.599	9.403	<.001	Accepted
R-square				
Teachers’ Technology Acceptance	0.36			

Model Fit Statistics (x^2^ = 210.616, df = 76, Ratio x^2^/df = 2.771; CFI = .969, TLI = .958, SRMR = .0508, and RMSEA = .076.)

## 6. Discussion

### 6.1 Middle leaders’ competency model in education 4.0 (MLCMEdu4.0)

In Education 4.0, critical thinking has become the primary competency for middle leadership [13]. This skill is essential for analyzing, evaluating, and synthesizing information, allowing middle leaders to tackle complex issues and make well-informed choices [7]. Research by [15] emphasizes the importance of strong analytical abilities in middle leaders for strategic planning and decision-making. The shift towards prioritizing critical thinking over instructional leadership or interpersonal skills reflects the increasing complexity of educational settings, which require leaders to handle ambiguity and make decisions based on data [12,61]. Middle leaders who consider various viewpoints contribute to organizational flexibility and readiness for unforeseen challenges [9,31,62].

The entrepreneurial constructs, with its high standardized estimate underscores the crucial role of innovation, risk-taking, and adaptability in middle leadership, particularly amidst ongoing educational changes. This represents a departure from conventional leadership paradigms, emphasizing the necessity for agile, adaptable leaders in the era of Education 4.0 [13,27]. The emphasis on adaptability and lifelong learning mirrors the changing landscape of school leadership, where flexibility and innovative thinking are essential for managing rapid transformations [31,63].

Middle leadership’s pivotal role in fostering collaboration is highlighted by this strong standardized estimate in Education 4.0. Consistent with previous studies [20,64], collaboration remains essential in Education 4.0. Middle leaders foster a supportive, cohesive environment, reducing stress and improving job satisfaction by promoting teamwork among teachers, encouraging cross-department initiatives, equitable resource sharing, and interdisciplinary learning [20]. They also engage external stakeholders, enhancing student learning [10]. Strong collaboration skills serve as a foundation for other leadership competencies, boosting effectiveness in communication, ethics, and instructional leadership [12,34,61].

The strong standardized estimate for Leading for Learning highlights its importance within the structural model of teachers’ technology acceptance. This finding indicates that instructional leadership is closely linked to fostering teachers’ willingness to adopt new technologies, which is an important step toward their effective use in the classroom. Middle leaders, drawing on their subject expertise, serve as credible mentors who nurture professional learning communities (PLCs). These PLCs are vital platforms for teacher growth and knowledge exchange [6,7,20]. Middle leaders cultivate environments that promote teacher collaboration, integrating the wisdom of experienced staff with the innovative ideas of newer educators. They also tailor their strategies to address diverse learning requirements [34].

Digital Dexterity in this study show high standardized estimate emphasizes its growing importance in effective middle leadership within educational contexts [3,13,27]. This finding aligns with recent research highlighting the need for technological competence, particularly in light of rapid digitalization and events like the COVID-19 pandemic [5]. While digital competency among leaders in schools has received moderate attention in research [65,66], studies specifically focusing on middle leaders’ digital dexterity are comparatively scarce. Recent studies [12,38] highlight the challenges middle leaders face in navigating digitalization, often constrained by limited skills, time, and resources in schools. To overcome these barriers and advance technological integration in education, middle leaders must demonstrate adaptability and strategic leadership.

Emotional Intelligence shows a significant but not top-ranking standardized estimate, highlighting its importance in leadership roles, particularly for interpersonal skills and self-awareness. This moderate loading aligns with [67], suggesting that emotional intelligence’s impact on transformational leadership and overall competency might be less pronounced than previously thought. This could be due to social desirability bias and its status as a foundational skill. However, it’s worth noting that earlier research, such as [15,30] often emphasized emotional intelligence as a crucial leadership competency. In the educational context, [7,9] mentioned that middle leaders are particularly adept at providing emotional support and appreciation, which is essential for fostering strong relationships within schools.

Management and Administration, with relatively lower standardized estimate, indicate that while these skills are essential for effective functioning, they don’t necessarily define exceptional leadership. Instead, they provide a foundational support for other leadership functions, particularly in critical times. This aligns with research by [7,24,68], which suggests that management tasks are often seen as supportive rather than primary drivers of leadership effectiveness. Middle leaders typically manage activities within their subject or department panels, handling operational tasks such as organizing meetings, developing yearly plans, managing documentation, and coordinating activities [6,11,18,69]. They also manage financial responsibilities like Per Capita Grants (PCG) for teaching and learning resources, though their authority is limited to panel-level decisions, with broader school management handled by senior leadership [14,15].

The relatively low standardized estimate for Communication and Ethics is surprising, given the traditional emphasis on strong communication in leadership roles. Middle leaders act as a bridge, with dual responsibilities in communication [6,8,12,69,70]: Downward communication – conveying information, feedback, and directives to teachers in a clear, supportive, and motivating manner; Upward communication – articulating teachers’ concern, ideas, and feedback to senior leaders with diplomacy and clarity. This might suggest that while communication and ethical considerations remain important, they are not seen as distinguishing factors for overall leadership competency in this model. Instead, they may be viewed as foundational skills that are expected rather than being standout competencies in Education 4.0.

Decision-making and Problem-solving has the lowest standardized estimate in this study, which is surprising given its traditionally crucial role in leadership. This contrasts with [13] School Leader Competency Model (SLCMEduc4.0), where decision-making held a higher estimate. However, that study focused on senior leaders, who have more autonomy in decision-making [15,33]. Middle leaders, on the other hand, engage in panel-level decision-making, often taking a collaborative approach by seeking input from teachers and making decisions collectively, consistent with recent findings by [10]. This distributed leadership approach significantly contributes to school improvement efforts [12,24].

### 6.2 Teachers’ technology acceptance (TTA)

Perceived Ease of Use emerges as the key factor indicating that teachers prioritize how easy and intuitive a technology is to use. [71] highlights that this time-saving aspect is crucial for technology acceptance, with PEU having a significant impact on adoption rates [72]. User-friendly tools boost confidence, reduce errors, and increase teachers’ comfort with technology adoption, regardless of age [73–76].

The high Perceived Usefulness suggests teachers are more willing to implement technologies they believe will enhance their job performance and effectiveness. Perceived Usefulness is critical for long-term technology adoption in schools [72,77]. Unlike Perceived Ease of Use, which has a more immediate impact, the benefits of usefulness often become more apparent over time as teachers experience advantages in their practices [47]. The impact of Perceived Usefulness on technology acceptance aligns with Rogers’ Diffusion of Innovations theory [78,79]. Early adopters, initially influenced by Perceived Ease of Use, demonstrate usefulness to others, gradually increasing its perceived value in the educational community.

Facilitation Condition shows a strong influence, highlighting the importance of organizational and technical support in technology adoption. This suggests teachers tend to use technology when they perceive they have the necessary resources, support, and infrastructure to do so effectively. [44,80] explained that ensuring access to up-to-date technology and support can significantly reduce adoption barriers. Regular training sessions, workshops, and access to instructional technology coaches can help teachers stay updated and feel more competent in using technology [81,82].

Technology Self-Efficacy has high standardized estimate, indicating its significant role in technology acceptance. This suggests that teachers’ belief in their capability to use technology efficiently is an important aspect in their openness to embrace emergent technological innovations. Teachers’ competence with technology positively influences their integration of it into teaching practices, with factors such as seeking assistance, managing effort, and maintaining a growth-oriented mindset, facilitating conditions, perceived usefulness, and ease of use directly affecting their willingness to continue using technology in education [76,83].

Subject Norm shows a moderately strong influence indicating that social influences and perceived expectations from peers, administrators, and the broader educational community play a notable role in teachers’ technology acceptance. However, its impact is moderated by several factors. Social influence appears to have less impact compared to other constructs, such as perceived ease of use or usefulness. The availability of support and infrastructure often outweighs social pressure in adoption decisions [84]. Teachers in this study may resist peer pressure, basing their decisions on personal experiences and professional judgment. This emphasis on individual decision-making in education could be making social norms less impactful in technology adoption [85,86]. These findings align with the observation that experienced teachers prioritize their own judgment, viewing themselves as classroom experts.

Technology Pedagogy and Content Knowledge (TPACK) has the lowest standardized estimate, yet still shows substantial influence. This suggests that while teachers’ ability to integrate technology with their pedagogical and content knowledge is important. [87] found TPACK’s impact on technology acceptance to be less pronounced than anticipated. This discrepancy may stem from technology integration often remaining superficial, primarily focused on presentation tools rather than deep integration into pedagogical practices. [88] supports this notion, showing that while teachers demonstrated higher competence in using technology during teaching processes, their ability to develop learning materials based on students’ needs was lacking.

### 6.3 Middle leaders’ competency in education 4.0 impacts on teachers’ technology acceptance

This study found a significant positive relationship between middle leaders’ competency and teachers’ technology acceptance in Malaysian TS25 schools. Results show a moderately strong effect (β = 0.599, p < .001), with middle leaders’ competency explaining 36% of the variance (R² = .36) in teachers’ acceptance of technology. This highlights the pivotal role middle leaders play in shaping teachers’ willingness to adopt educational technologies.

At the same time, the findings also remind us that technology acceptance is a multifaceted phenomenon. A substantial portion of the variance (64%) remains unexplained by this model. This indicates that while middle leader competency is an important predictor, teachers’ acceptance of technology is also influenced by a wider ecosystem of factors. Likely contributors include systemic elements such as national education policies and principal-level leadership; organizational supports like ICT infrastructure, access to digital resources, and professional development opportunities; as well as teacher-level factors including prior digital experience, subject area demands, pedagogical beliefs, and personal interest in technology.

Given the cross-sectional and self-report design, reciprocal explanations cannot be ruled out. For example, teachers who are already more accepting of technology may also rate middle leadership more favorably and residual common-method bias may remain despite our validity checks.

The substantial impact of middle leaders’ competency can be understood through their influence on key aspects of technology acceptance:

i. Perceived Usefulness: Middle leaders demonstrate technology’s value through active use and early adoption, showcasing tangible benefits and efficiency gains. This proactive approach reduces adoption barriers by addressing challenges and presenting refined applications [6,9,18]. By integrating technology into daily practices, they positively influence teachers’ perceived usefulness of technology [11,13].
ii. Perceived Ease of Use: By providing readily available technical support and tailored training, middle leaders significantly reduce technology adoption barriers [89,90]. Middle leaders can break down complex technologies into user-friendly components, making them accessible to all teachers [91,92].
iii. Subject Norm: Middle leaders shape the school’s technology culture by consistently using and promoting digital tools. They facilitate technology-driven collaborations and professional development, establishing technology use as an expected professional practice [18,67]. Their influence extends to managing technology integration during difficult periods, including the transition to remote learning amid the COVID-19 pandemic. [35,70].
iv. Facilitation Condition: By leveraging internal expertise and external resources, middle leaders create an environment conducive to technology acceptance [2,12]. They connect tech-savvy teachers with those needing support, fostering a collaborative culture of technology adoption [93]. Middle leaders ensure accessibility of user-friendly technology resources and actively support technology adoption by encouraging teacher leadership [92].
v. Technology Self-Efficacy: Middle leaders enhance teachers’ technology self-efficacy through hands-on support and emotional encouragement [92,94]. They create a safe environment for experimentation where questions are welcomed and mistakes are seen as learning opportunities [95]. This approach builds confidence in teachers’ ability to master new technologies and promotes a growth mindset [96].
vi. Technology Pedagogy and Content Knowledge: Through organizing Professional Learning Communities, middle leaders enhance teachers’ Technological Pedagogical Content Knowledge (TPACK) [92]. They actively share new apps, digital resources, and online tools relevant to different subject areas [94]. By conducting targeted workshops and professional development sessions, they foster continuous improvement in technology integration across subjects [9,10].

## 7. Limitations

This study has several limitations that should be acknowledged. First, its focus on urban primary schools in Kuala Lumpur restricts generalizability to the wider Malaysian educational context, particularly rural or secondary schools that may face different challenges. Second, the cross-sectional design offers only a snapshot of the relationship between middle leaders’ competency and teachers’ technology acceptance, without capturing how these dynamics may evolve over time. Third, although the sample of 302 teachers satisfied several established guidelines for SEM, it remains modest for a model of this complexity. While the sample enabled stable estimation, larger samples would strengthen statistical power, reduce the likelihood of Type II errors, and enhance generalizability. Moreover, several post-hoc model modifications, including correlated error terms, were introduced to achieve good fit. While such adjustments are accepted practice, they may risk overfitting the model to this specific sample, potentially limiting external validity. Fourth, the explanatory power of the model is moderate (R² = .36). While this demonstrates a meaningful association, it also suggests that important determinants remain outside the scope of the study. Factors such as principal leadership, school ICT policy, infrastructure availability, training intensity, and broader systemic or cultural influences likely account for additional variance in technology acceptance. Finally, reliance on teachers’ perceptions of middle leaders’ competencies introduces subjectivity and may not fully reflect actual leadership practices. Self-report measures also raise the possibility of common-method bias and reciprocal effects. For example, teachers already more accepting of technology might view their leaders more favorably. Taken together, these limitations indicate that the present findings should be interpreted as preliminary evidence of the role of middle leaders in shaping technology acceptance. Future research would benefit from larger, more diverse samples, longitudinal or multilevel designs, and the inclusion of additional school- and system-level factors to provide a more comprehensive understanding of the phenomenon.

## 8. Recommendations for practice

To enhance middle leaders’ impact on technology acceptance, schools should implement targeted professional development programs focusing on crucial competencies like digital dexterity, decision-making, and problem-solving. The position and functions of middle leaders should be clarified and potentially expanded to better leverage their influence. Schools should foster a collaborative culture that promotes peer learning and mentoring in technology integration. Developing school-wide strategies for technology integration that explicitly involve middle leaders as key facilitators is crucial. Establishing robust support systems, including technical support and resource allocation, is essential for both middle leaders and teachers in their efforts to integrate technology effectively.

## 9. Future research recommendations

Future studies should expand beyond urban primary schools to include diverse educational settings, such as rural and secondary schools across Malaysia. This would provide richer insights into the varied contexts in which middle leadership shapes technology acceptance. Comparative studies between different types of schools could also help identify best practices that are transferable across settings. Longitudinal research is needed to capture how the relationship between middle leaders’ competencies and teachers’ technology acceptance evolves over time. In addition, recruiting larger, multi-site samples planned a priori against model complexity (e.g., parameter count) would improve statistical power, model stability, and generalizability of SEM results. Exploring the interplay between sample size and model complexity could also offer valuable methodological insights for future educational research. Methodologically, mixed-methods approaches that combine surveys with in-depth qualitative work could uncover the mechanisms through which middle leadership interacts with senior leadership, school culture, infrastructure, and system-level policies to shape teachers’ acceptance. Cross-cultural comparative research and studies examining specific types of educational technologies would further enrich the evidence base. Finally, future research could revisit the psychometric foundations of the instruments used. Applying data-driven approaches such as Exploratory Factor Analysis (EFA) or Principal Component Analysis (PCA) alongside confirmatory methods would help verify whether the factor structures established in other contexts hold true for Malaysian teachers. This would not only validate the instruments across cultures but also refine their applicability to different educational systems.

## 10. Conclusion

As a conclusion, this study illuminates the significant influence of middle leaders’ competencies on teachers’ technology acceptance in Malaysian TS25 primary schools within the context of Education 4.0. The research underscores the crucial role of middle leaders as catalysts for technological integration and educational innovation. By leveraging their unique position between senior management and teaching staff, middle leaders can effectively bridge policy intentions with classroom realities, particularly in the adoption of new technologies. These findings emphasize the importance of developing comprehensive professional development programs that enhance middle leaders’ competencies across various domains. Despite limitations in scope and methodology, this study provides valuable insights for educational policy and practice, offering a foundation for future research on middle leadership’s role in driving educational transformation and technology integration in diverse school settings.
